# Derivation and Internal Validation of a Mortality Prognostication Machine Learning Model in Ebola Virus Disease Based on Iterative Point-of-Care Biomarkers

**DOI:** 10.1093/ofid/ofad689

**Published:** 2024-01-05

**Authors:** Courtney J Bearnot, Eta N Mbong, Rigo F Muhayangabo, Razia Laghari, Kelsey Butler, Monique Gainey, Shiromi M Perera, Ian C Michelow, Oliver Y Tang, Adam C Levine, Andrés Colubri, Adam R Aluisio

**Affiliations:** Department of Emergency Medicine, Alpert Medical School of Brown University, Providence, Rhode Island, USA; International Medical Corps, Goma, Democratic Republic of Congo; International Medical Corps, Goma, Democratic Republic of Congo; International Medical Corps, Goma, Democratic Republic of Congo; Program in Bioinformatics and Integrative Biology, University of Massachusetts Chan Medical School, Worcester, Massachusetts, USA; Rhode Island Hospital, Providence, Rhode Island, USA; International Medical Corps, Washington, DC, USA; Division of Infectious Diseases and Immunology, Department of Pediatrics, School of Medicine, University of Connecticut, Farmington, Connecticut, USA; Department of Emergency Medicine, Alpert Medical School of Brown University, Providence, Rhode Island, USA; Department of Neurosurgery, University of Pittsburgh, Pittsburgh, Pennsylvania, USA; Department of Emergency Medicine, Alpert Medical School of Brown University, Providence, Rhode Island, USA; Program in Bioinformatics and Integrative Biology, University of Massachusetts Chan Medical School, Worcester, Massachusetts, USA; Department of Emergency Medicine, Alpert Medical School of Brown University, Providence, Rhode Island, USA

**Keywords:** biomarker, Ebola virus disease, humanitarian response, machine learning, point-of-care testing

## Abstract

**Background:**

Although multiple prognostic models exist for Ebola virus disease mortality, few incorporate biomarkers, and none has used longitudinal point-of-care serum testing throughout Ebola treatment center care.

**Methods:**

This retrospective study evaluated adult patients with Ebola virus disease during the 10th outbreak in the Democratic Republic of Congo. Ebola virus cycle threshold (Ct; based on reverse transcriptase polymerase chain reaction) and point-of-care serum biomarker values were collected throughout Ebola treatment center care. Four iterative machine learning models were created for prognosis of mortality. The base model used age and admission Ct as predictors. Ct and biomarkers from treatment days 1 and 2, days 3 and 4, and days 5 and 6 associated with mortality were iteratively added to the model to yield mortality risk estimates. Receiver operating characteristic curves for each iteration provided period-specific areas under curve with 95% CIs.

**Results:**

Of 310 cases positive for Ebola virus disease, mortality occurred in 46.5%. Biomarkers predictive of mortality were elevated creatinine kinase, aspartate aminotransferase, blood urea nitrogen (BUN), alanine aminotransferase, and potassium; low albumin during days 1 and 2; elevated C-reactive protein, BUN, and potassium during days 3 and 4; and elevated C-reactive protein and BUN during days 5 and 6. The area under curve substantially improved with each iteration: base model, 0.74 (95% CI, .69–.80); days 1 and 2, 0.84 (95% CI, .73–.94); days 3 and 4, 0.94 (95% CI, .88–1.0); and days 5 and 6, 0.96 (95% CI, .90–1.0).

**Conclusions:**

This is the first study to utilize iterative point-of-care biomarkers to derive dynamic prognostic mortality models. This novel approach demonstrates that utilizing biomarkers drastically improved prognostication up to 6 days into patient care.

## Teaser

This is the first study to utilize iterative point-of-care biomarkers to derive a dynamic prognostic mortality model for Ebola virus disease. This model overcomes the limitations of previous models by extending prognostication to day 6 of patient care.

The 2014–2016 West Africa Ebola virus disease (EVD) outbreak was the most geographically widespread and deadliest to date, the scale and duration of which prompted significant research on the natural history and outcomes of the disease [1]. Several studies found that Ebola virus cycle threshold (Ct) based on reverse transcriptase polymerase chain reaction (PCR)—a value inversely proportional to viral load—was important in mortality prognostication [2, 3]. Since then, multiple clinical prognostic models have used Ct, epidemiologic, and symptom-based data to identify risk of death and inform care delivery for patients infected with EVD [4–7]. A small number of studies have reported associations between EVD mortality and elevations in biomarkers such as C-reactive protein (CRP), blood urea nitrogen (BUN), creatinine, aspartate aminotransferase (AST), and alanine aminotransferase (ALT) [3, 8–11]. However, during the West Africa outbreak, laboratory facilities were scarce, and serum biomarker testing was not available at most Ebola treatment centers (ETCs). Therefore, the incorporation of biomarker data into prognostic models has been limited.

The 10th EVD outbreak in the Democratic Republic of Congo (DRC) from 2018 to 2020 was the second deadliest, with 2287 deaths and 3470 confirmed cases [12]. Point-of-care (POC) serum testing was more widely available during this outbreak, allowing the integration of biomarkers into prognostic scores for adult and pediatric populations with EVD [13, 14]. Although these scores have potential utility in informing patient care, the research to date is limited by the use of biomarker data from the first 48 hours of ETC admission. These single–time point approaches are unable to integrate changes in clinical states and biomarkers over the patient's treatment course. Given this, the current study aimed to derive a dynamic biomarker-based tool for iterative mortality prognostication by using machine learning (ML) models among persons with EVD, with data collected from the 2018–2020 DRC outbreak.

## METHODS

### Study Participants and Setting

This retrospective cohort study included patients aged ≥18 years admitted to International Medical Corps' Mangina ETC with laboratory-confirmed EVD from December 2018 to January 2020. The Mangina ETC, located in the North Kivu province, served the catchment areas of the North Kivu and Ituri provinces. It had a capacity of 102 beds and was staffed by 17 physicians and 43 nurses. Patients were excluded if their survival outcome or Ct was not recorded.

### Patient Consent Statement

The project received a human research exemption from the institutional review board at Rhode Island Hospital (Lifespan Health System; No. 1527875). All research was performed in accordance with DRC government regulations. No additional local approval was required.

### Clinical Approach and Biomarker Testing

ETC staff screened all persons according to World Health Organization and Médecins Sans Frontières guidelines [15, 16]. Patients with PCR-confirmed EVD were admitted directly to the confirmed ward. Patients with suspected cases were admitted to a separate ward, where they underwent serologic testing with reverse transcriptase PCR (Xpert Ebola Assay; Cepheid). If results were negative, patients were tested again after 72 hours and then discharged if the second test result was negative. If the result was positive, patients were admitted to the confirmed ward. The Xpert Ebola Assay was also used to determine Ct, the minimum number of PCR cycles required to detect the target RNA. Ct is an inversely proportional proxy for viral load; a cutoff >40 cycles indicated a negative test result. All EVD laboratory testing was conducted by the Institut National de Recherche Biómedicale, DRC.

POC serum biomarker testing was available for use at the discretion of the clinical providers. POC testing was performed with the Piccolo Xpress chemistry analyzer (Abaxis) and Piccolo AmLyte 13 cartridges, which measure ALT, albumin, amylase, AST, BUN, calcium, CRP, creatinine, creatinine kinase, glucose, potassium, sodium, and total bilirubin [17].

Medical records were maintained in hard copy for each patient. At ETC triage, information was collected on demographics, symptoms, symptom onset, and self-reported vaccination status. Patients were evaluated daily by medical staff, and clinical information was documented at each assessment. Ct and POC biomarker testing were documented as utilized.

Clinical records were retrospectively digitalized into a secure server according to previously validated methods [18], deidentified, and manually extracted by trained research staff who were blinded to the study aims. Data quality assurance was assessed by double entering 15% of records. The double-entry audit found 97.3% concordance between the data sets.

### Statistical Analysis

Demographic data, self-reported vaccination status, symptoms at triage, and mortality outcome were summarized by median and IQR for continuous variables and frequency and percentage for categorical variables. To ensure sufficient data for modeling, biomarker data were combined into 2-day periods: treatment days 1 and 2 (D_1,2_), days 3 and 4 (D_3,4_), days 5 and 6 (D_5,6_). Treatment day 1 referred to the calendar day of a patient's ETC admission. Modeling beyond treatment day 6 was not possible due to high class imbalance, as most deaths occurred in the first week of care. Biomarker distribution across D_1,2_, D_3,4_, and D_5,6_ was summarized by median and IQR. Consistent with prior literature, high viral load was defined as Ct ≤22 [19]. As sodium, potassium, calcium, and glucose can have deleterious effects at elevated and reduced values, they were categorized as high, normal, and low based on Piccolo AmLyte 13 reference ranges [17]. Biomarkers were compared by mortality outcome according to Pearson chi-square or Fisher exact test for categorical variables and by Mann-Whitney or *t* test for continuous variables, as appropriate.

An iterative series of logistic regression prognostic ML models for the outcome of mortality during ETC care were created with data from patients with recorded values for candidate predictors for a given period. The ML model cannot hold an infinite number of variables. Therefore, biomarkers to enter the model were chosen a priori according to clinical expertise and statistically significant differences between survivors and nonsurvivors. The serial ML models used the risk profile from the preceding model to provide an updated risk prediction and, in turn, inform subsequent predictions. This approach provided a more temporally dynamic output to account for changes in clinical status over time and addressed the limitations of using data from only initial presentation. Building from prior work, a base model was used with predictors of age and Ct value at ETC admission, which were modeled as continuous variables [6]. In addition to the risk prediction from the preceding model, all Ct and POC biomarkers measured within 2 days from the preceding prognostic model were iteratively incorporated to construct updated models for each of the 3 periods. Transformations were applied to certain biomarkers to account for their clinical impacts. For example, potassium was converted into a unimodal variable by subtracting the mean of the normal range and taking the absolute value of this difference, while albumin was coded inversely by subtracting each value from the maximum observed among all samples.

The base model was augmented with biomarkers from D_1,2_, D_3,4_, and D_5,6_ and Ct values from D_3,4_, and D_5,6_ according to a validated protocol that recalibrated the base model and incorporated additional predictors simultaneously by fitting a new model with the linear predictors of the base model and the additional biomarkers [20]. Biomarkers were selected per elastic net regularization, which combines the Lasso and Ridge regression methods to handle multicollinearity [13, 21]. The threshold for variable inclusion was set at 70% to exclude variables with weak or inconsistent effects. This threshold also ensured that no pair of variables had a Pearson correlation coefficient >0.65, therefore reducing the potential for model issues due to collinearity [22]. The updated model that resulted from augmenting the base model with the selected biomarkers from D_1,2_ was subsequently augmented with the Ct values and selected biomarkers from D_3,4_, and again augmented with those from D_5,6_. Bootstrap resampling was used for internal validation of each recursive model. Discrimination was evaluated by optimism-corrected area under curve (AUC) of the receiver operating characteristic (ROC) curves, while calibration was assessed through a calibration plot comparing predicted probabilities with observed outcome [23]. ROC curves with AUC and associated 95% CIs were derived for each 2-day clinical period of interest. The ROC curves were generated by the pROC package with R Statistical Software (version 4.3.2; R Core Team).

A sensitivity analysis was conducted to assess the iterative prognostic model results without longitudinal Ct data, as Ct values are not a POC biomarker. This analysis followed the same methodology as the primary model but eliminated Ct as a variable from all but the base model. The base model was augmented with D_1,2_ biomarkers selected through elastic net regularization. Selected biomarkers from D_3,4_ were subsequently incorporated, followed by those from D_5,6_. To evaluate the potential impact of EVD-specific therapeutics, a second sensitivity analysis was performed among the subset of patients who received mAb114 or REGN-EB3 following the same methodology.

## RESULTS

### Cohort Characteristics

**Figure 1.**
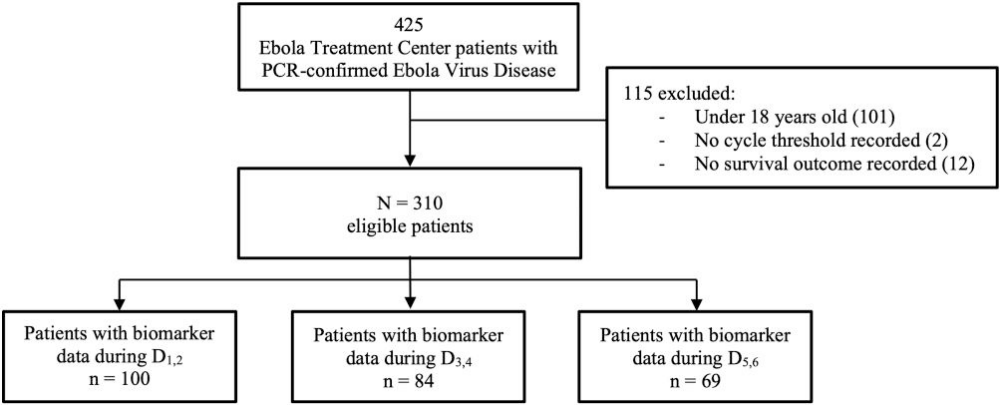
Flowchart with exclusion criteria for the Mangina Ebola virus disease patient cohort. D_1–6_, treatment days 1–6; PCR, polymerase chain reaction.

From December 2018 to January 2020, 425 patients with PCR-confirmed EVD were admitted to the ETC. The study included 310 patients after excluding 101 patients <18 years old, 2 with no documented Ct value, and 12 with no documented survival outcome. Biomarker data were available for 100 patients during D_1,2_, 84 during D_3,4_, and 69 during D_5,6_ (Figure 1). There were no statistically significant differences in sex, age, self-reported vaccination status, or symptoms at triage (Supplementary Table 2).

The median age was 32 years (IQR, 24–46), and 43.2% of patients were male. Patients were admitted a median 4 days (IQR, 2–6) after symptom onset. Common symptoms at admission were fatigue (84.1%), anorexia (74.8%), subjective fever (70.3%), headache (67.3%), arthralgia (62.9%), and abdominal pain (57.1%). Self-reported vaccination with the rVSV-ZEBOV vaccine was 44.0%, and 71.9% of patients reported having received the vaccine within 14 days of ETC admission. The Mangina ETC was a Pamoja Tulinde Maisha (PALM) trial study site. Patients were randomized to receive remdesivir (20.3%), REGN-EB3 (30.3%), mAb114 (30.6%), or ZMapp (3.2%), and 15.5% did not receive any of these therapies. Mortality occurred in 46.5% of cases (Table 1). High viral load was observed in 73.8% of those who died, as opposed to 21.8% of survivors (*P* < .0001).

**Table 1.** Cohort Characteristics (N = 310)

Variable	No. (%)
Sex	
Male	134 (43.2)
Female	176 (56.8)
Age, y, median (IQR)	32 (24–46)
Vaccinated with rVSV-ZEBOV vaccine	135 (44.0)
Time symptom onset to presentation, d, median (IQR)	4 (2–6)
Symptoms reported at admission^a^	
Fatigue	260 (84.1)
Anorexia	232 (74.8)
Subjective fever	218 (70.3)
Headache	208 (67.3)
Arthralgia	195 (62.9)
Abdominal pain	177 (57.1)
Myalgia	175 (56.5)
Nausea	153 (49.4)
Conjunctivitis	150 (48.5)
Diarrhea	149 (48.2)
Chest pain	113 (36.6)
Cough	84 (27.2)
Dysphagia	67 (21.6)
Abnormal bleeding^b^	59 (19.1)
Sore throat	57 (18.5)
Dyspnea	48 (15.5)
Hiccup	17 (5.5)
Coma	14 (4.5)
Rash	13 (4.2)
Jaundice	11 (3.6)
Confusion	11 (3.6)
Eye pain	8 (2.6)

^a^Percentages may sum to >100% due to multiple reported symptoms per person.

^b^Abnormal bleeding—including hematochezia, bleeding gums, nonmenstrual vaginal bleeding, hematemesis, and epistaxis—was aggregated into a single variable.

### POC Biomarker Profile

**Figure 2.**
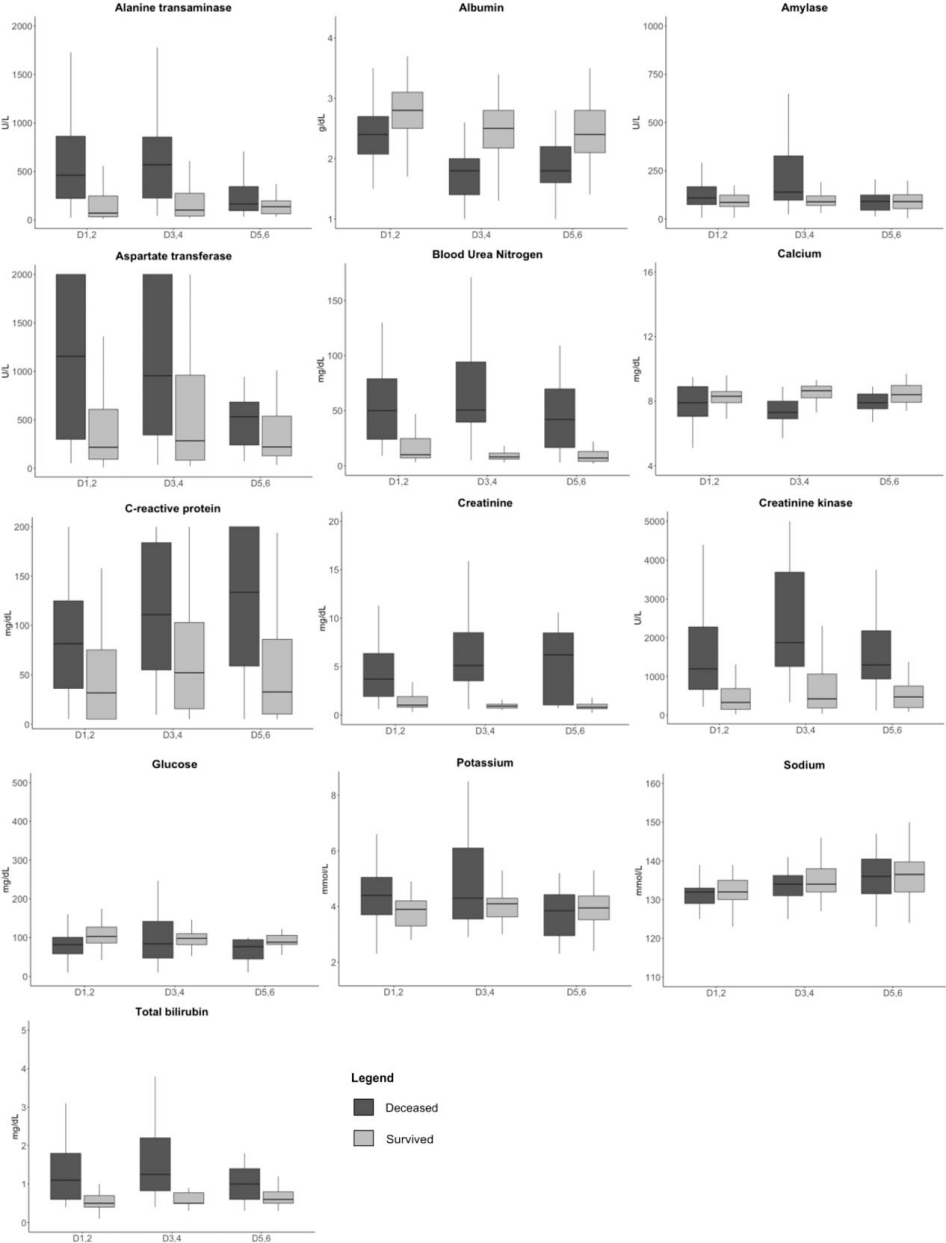
Biomarkers on treatment day_1,2_, day_3,4_, and day_5,6_. Line, median; box, IQR; error bars, 95% CI.

Summary biomarker data were stratified by mortality across D_1,2_, D_3,4_, and D_5,6_ (Figure 2). Hepatic biomarkers, including total bilirubin, AST, and ALT, were significantly higher among nonsurvivors during D_1,2_, and D_3,4_ but were not significantly different during D_5,6_. AST showed greater variance vs ALT in survivors and nonsurvivors. Primary renal biomarkers, including BUN and creatinine, were significantly higher among nonsurvivors during all 3 periods. Albumin was significantly lower among nonsurvivors in all periods. CRP was significantly higher in nonsurvivors during all 3 periods and showed an upward trend among nonsurvivors between D_1,2_ and D_5,6_. In comparison, CRP in survivors initially increased between D_1,2_ and D_3,4_ but then decreased between D_3,4_ and D_5,6_.

Except for sodium, which was similar between groups during all periods, nonsurvivors were more likely to have electrolyte abnormalities than survivors (Supplementary Table 1). When compared with survivors, nonsurvivors experienced more hyperkalemia (D_1,2_, 22.6% vs 1.8%, *P* = .0051; D_3,4_, 37.0% vs 6.5%, *P* = .0019; D_5,6_, 18.8% vs 4.0%, *P* = .069) and hypocalcemia (D_1,2_, 56.4% vs 29.3%, *P* = .024; D_3,4_, 69.7% vs 16.7%, *P* < .0001; D_5,6_, 50.0% vs 26.0%, *P* = .081), though statistical significance was not reached during D_5,6_. Additionally, nonsurvivors had a higher frequency of hypoglycemia (D_1,2_, 36.8% vs 12.1%, *P* = .011; D_3,4_, 42.4% vs 16.7%, *P* = .0011; D_5,6_, 50.0% vs 18.0%, *P* = .025).

### Prognostic Models

**Figure 3.**
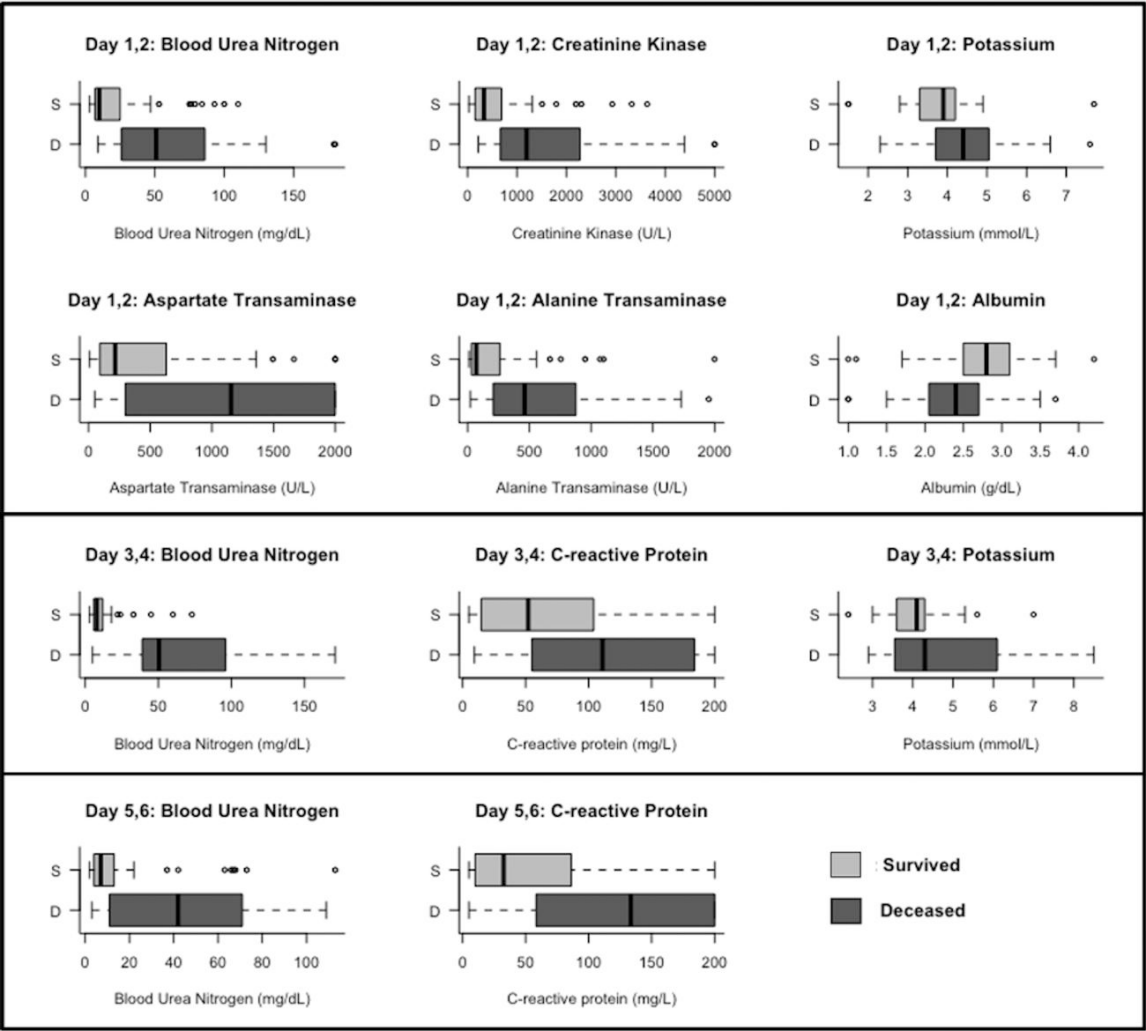
Biomarkers included in mortality prognostic models based on treatment day_1,2_, day_3,4_, and day_5,6_. Line, median; box, IQR; error bars, 95% CI; circles, outliers.

Figure 3 displays distributions of the biomarkers associated with mortality identified through elastic net regularization regression. Significant predictors of mortality were elevated creatinine kinase, AST, BUN, ALT, and potassium; hypoalbuminemia during D_1,2_; elevated CRP, BUN, and potassium during D_3,4_; and elevated CRP and BUN during D_5,6_.

**Figure 4.**
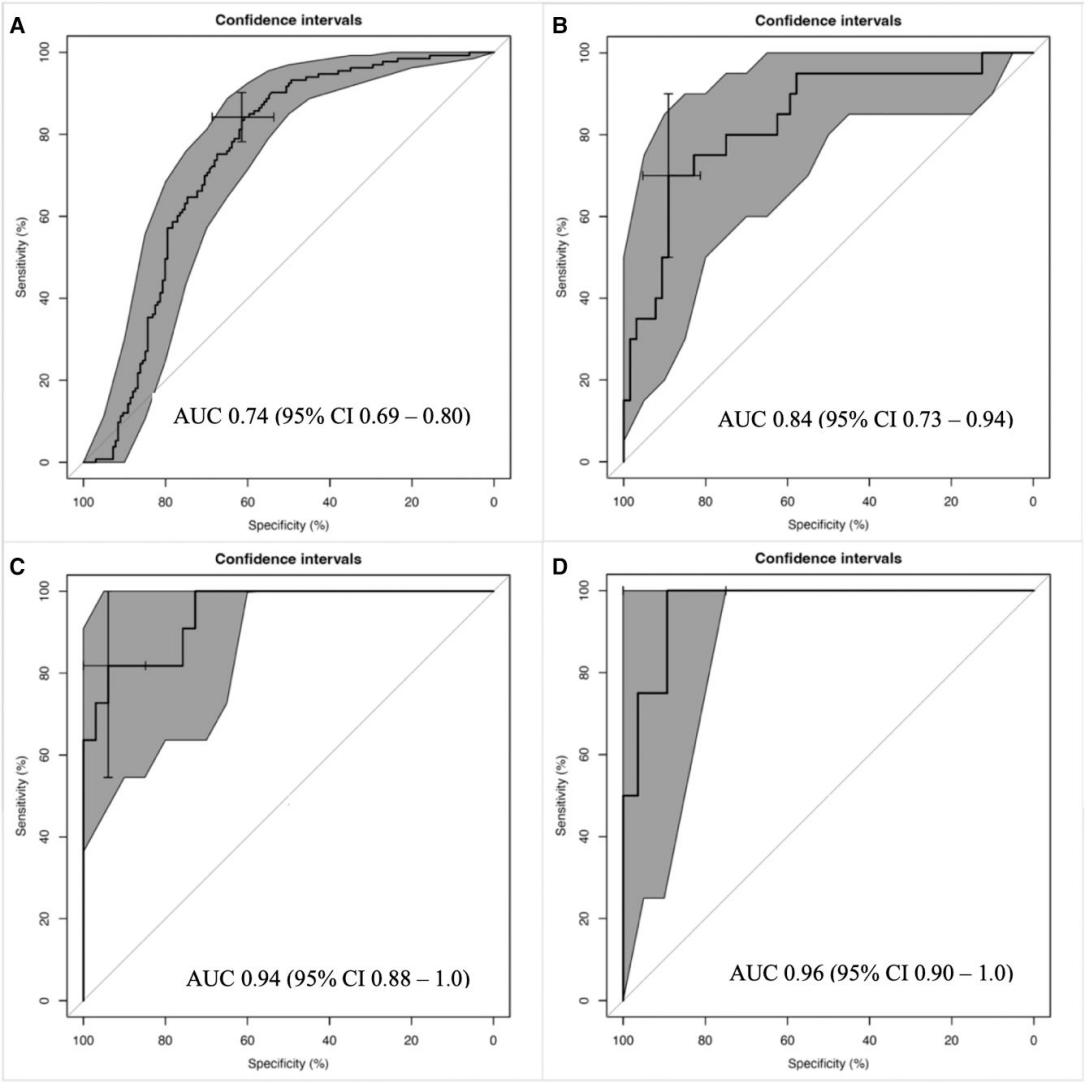
Receiver operating characteristic curves for the mortality prognostic models based on iterative biomarker data: *A*, base model including age and cycle threshold; *B*, treatment day_1,2_; *C*, treatment day_3,4_; and *D*, treatment day_5,6_. Central black line, curve; gray shaded area, 95% CI. AUC, area under the curve.

ROC curves for the iterative ML prognostic models are presented in Figure 4. The base model, including only age and Ct on admission, achieved an AUC of 0.74 (95% CI, .69–.80; Figure 4*A*). The D_1,2_ model, incorporating D_1,2_ biomarkers into the base model, improved the AUC to 0.84 (95% CI, .73–.94; Figure 4*B*). The D_3,4_ model further augmented the D_1,2_ model by incorporating D_3,4_ Ct and biomarkers, resulting in an AUC of 0.94 (95% CI, .88–1.0; Figure 4*C*). The D_5,6_ model incorporated all prior data as well as D_5,6_ Ct and biomarkers, achieving an AUC of 0.96 (95% CI, .90–1.0; Figure 4*D*). Calibration plots demonstrated agreement between the predicted and observed risk, with a tendency for risk overestimation (Supplementary Figure 1).

In the sensitivity analysis, biomarkers associated with mortality remained consistent with the primary model for each period. For D_1,2_, the AUC remained 0.84 (95% CI, .73–.94; Supplementary Figure 2*B*) since it already incorporated Ct on admission. Augmenting the D_1,2_ model with D_3,4_ biomarker values improved the AUC to 0.87 (95% CI, .72–1.0; Supplementary Figure 2*C*), and further incorporating D_5,6_ biomarkers values resulted in an AUC of 0.95 (95% CI, .87–1.0; Supplementary Figure 2*D*). Calibration curves for the sensitivity models are displayed in Supplementary Figure 3. Furthermore, the sensitivity analysis of the subset of patients who received REGN-EB3 or mAb114 revealed AUCs similar to the primary model (Supplementary Figure 4); however, the data were too sparse to perform the D_5,6_ model analysis.

## DISCUSSION

This study is the first to utilize iterative dynamic biomarker data from an outbreak response setting in a ML model for EVD mortality prognostication. This novel approach demonstrates that the addition of easily attainable POC biomarker data significantly improved the ability to identify patients at higher risk for mortality up to 6 days into patient care. In the era of increased availability of POC testing and continued limited access to EVD therapeutics [24, 25], these data suggest that inclusion of POC biomarkers may have clinical utility in identifying patients who are the most vulnerable and informing EVD response care. As the current analysis is a derivation of this potentially beneficial approach, further research with external validation and implementation assessments is needed to properly inform clinical application.

Although prior prognostic models have been reported, they are limited to biomarkers collected in the first 48 hours of ETC admission and lack data on the temporal variability as it relates to EVD mortality [4–7]. While early identification and prognostication are important, the ability to triage patients and allocate resources throughout their ETC admission would be beneficial and may improve clinical outcomes. The current iterative model is the first to include biomarker data past the first 48 hours, thereby addressing that limitation, and demonstrates an improvement in predictive accuracy over the course of care. The sensitivity analysis showed that the model is robust and was not substantially affected when Ct from D_3,4_ and D_5,6_ was eliminated. As PCR is a more intensive assay to complete, the results of the sensitivity analysis further support the pragmatic utility of POC biomarkers. The longitudinal modeling approach provides the advantage of allowing for the reevaluation of a patient's mortality risk over time during EVD treatment, and it suggests that the utilization of iterative ML model architecture could provide clinical prediction models that leverage more objective predictive parameters from biomarker data. Given the complexity of ML modeling over multiple periods, further work to develop digital health interfaces that are accessible and acceptable to health care providers is needed.

The temporal variation of biomarkers found to be associated with mortality in the current study is consistent with clinical reasoning and biologic plausibility. The D_1,2_ hepatic and renal dysfunction associated with mortality is well supported by prior research [3, 8–10]. Although AST had a larger variance than ALT in the population studied, the median AST value was more than twice as high as ALT, which has been described in EVD [11]. As ALT is primarily present in the hepatic tissue but AST also exists in heart, muscle, kidney, brain, pancreas, and lung tissue, this difference may reflect systemic cellular damage instead of direct hepatotoxicity of Ebola virus. BUN is the only variable that remained a constant predictor during the 3 prognostic periods and may represent a combination of volume contraction as well as systemic protein breakdown in the setting of inflammatory cytokine surge that is seen in EVD [26]. While the sensitivity analysis demonstrated stability of the biomarkers associated with mortality during each time point, it is possible that variations in specific prognostic biomarkers could exist according to individual patient characteristics. As such, future research to validate the iterative modeling is needed to confirm the biomarker temporal profile in EVD.

Significant advancements have been made in EVD therapeutics, yet access to these therapies in low-resource settings remains limited such that patient triage and resource management continues to be an essential element of outbreak response [25]. The World Health Organization guidelines for EVD therapeutics recommend treatment with mAb114 or REGN-EB3 for patients with PCR-confirmed EVD, as these therapies have shown to reduce EVD mortality [27, 28]. Nevertheless, aside from approval for compassionate use, neither therapy is approved for therapeutic use in the DRC, and manufacturers of the recommended treatments do not currently have plans to obtain licensure in African countries [24]. In this context, the stratification of patients with EVD to most accurately identify those at the highest risk of a poor outcome is crucial for providing optimal care and ensuring the most impactful resource allocations. This derived iterative POC biomarker model provides dynamic risk stratification and may represent a significant step toward improving care for patients with EVD, particularly when supply chains for therapeutics are constrained and dictate the need to focus treatments to those at highest risk for mortality.

### Limitations

Certain limitations should be considered when interpreting the current study. The lack of standardized biomarker collection may have introduced selection bias. However, testing was performed as deemed indicated by clinical providers and is representative of actual operating conditions in outbreak response care. Additionally, patients who died early after admission may not have had biomarker samples collected, and this could have introduced survival bias. Yet, one of the strengths of the iterative modeling approach is that patients did not require data from each time point to contribute to the model. The Mangina ETC was a PALM trial research site, and approximately 30% of patients received mAb114 or REGN-EB3. Furthermore, 44.0% self-reported having received the rVSV-ZEBOV vaccine. It was not possible to verify vaccination status, although >70% of patients reported that they had received the vaccine within 2 weeks of presentation to the ETC, which suggests that many may not have had sufficient time to mount protective immunity. Although the model did not account for patient-level pharmacotherapies and their potential impact on biomarker variations [29], none of the PALM trial therapies were shown to significantly affect viral clearance [28]. The incorporation of physiologic profiles over time may inherently account for such effects. Last, external validation and implementation research is required to develop digital health interfaces for future application of the prognostic tool in response efforts.

## CONCLUSION

The findings from the current study demonstrate that the POC biomarker-based iterative prognostic model yielded enhanced identification of patients with EVD who were at high risk for mortality up to 6 days into patient care. Given the legitimate limitations regarding availability of EVD-specific pharmacologic treatments in response settings, identification of patients' risk profiles over the duration of their treatment course is important to maximize resource allocation and minimize mortality. If future work validates the current findings, inclusion of POC biomarkers may have substantial clinical utility in informing EVD response care.

## Supplementary Material

ofad689_Supplementary_Data

## Supplementary Data

Supplementary materials are available at *Open Forum Infectious Diseases* online. Consisting of data provided by the authors to benefit the reader, the posted materials are not copyedited and are the sole responsibility of the authors, so questions or comments should be addressed to the corresponding author.

## Notes

***Author contributions.*** R. L., E. N. M., R. F. M., O. Y. T., A. C. L., and A. R. A. conceptualized the study. E. N. M., K. B., I. C. M., O. Y. T., A. C. L., A. C., and A. R. A. designed the study methodology. M. G. led project administration. R. L., E. N. M. , R. F. M., M. G., and S. M. P. conducted data collection. C. J. B., O. Y. T., K. B., and A. C. analyzed the data. A. C. and A. R. A. supervised the data analysis. C. J. B., A. C., A. R. A., and I. C. M. interpreted the data. C. J. B., A. C., and A. R. A. designed the data visualization. C. J. B. prepared the initial draft of the manuscript and all authors reviewed and edited the manuscript. All authors approved the final version of the manuscript. All authors had full access to all the data in the study and had final responsibility for the decision to submit for publication.

***Data sharing statement.*** Deidentified participant data with associated analysis syntax and information on the analytic protocol are available without restriction at https://github.com/colabobio/ebola-iterative-poc-biomarkers. Any queries on the data should be directed to the corresponding author.

***Disclaimer.*** The funding source had no role in the study design; in data collection, analysis, or interpretation; in manuscript writing; or in the decision to submit the work for publication.

***Financial support*.** This work was supported in part by the National Institute of Allergy and Infectious Diseases at the National Institutes of Health (R25AI140490 to O. Y. T.).
