# Supplementary material for: Derivation and Internal Validation of a Mortality Prognostication Machine Learning Model in Ebola Virus Disease Based on Iterative Point-of-Care Biomarkers
Source: Open Forum Infect Dis. 2024 Jan 5;11(2):ofad689. doi: 10.1093/ofid/ofad689 (PMC10878059; doi:10.1093/ofid/ofad689)
Supplement: ofad689_Supplementary_Data [file ofad689_supplementary_data.zip › supplements.docx]

**Figure S1.** Calibration curves for the mortality prognostic model using iterative biomarker data for base model including age and Ct (A), treatment day 1,2 (D_1,2_) model (B), treatment day 3,4 (D_3,4_) model (C), and treatment day 5,6 (D_5,6_) model (D). In the calibration plot, the dots represent the mean estimate of the observed probability for each 10% bin of predicted probability (with probability being risk of mortality), the vertical lines passing through each dot are the corresponding confidence intervals for the observed probability, the dashed line is the best linear fit passage through the mean values, and the solid curved line is the LOESS curve fitting all of the individual observed/predicted pairs in the data.


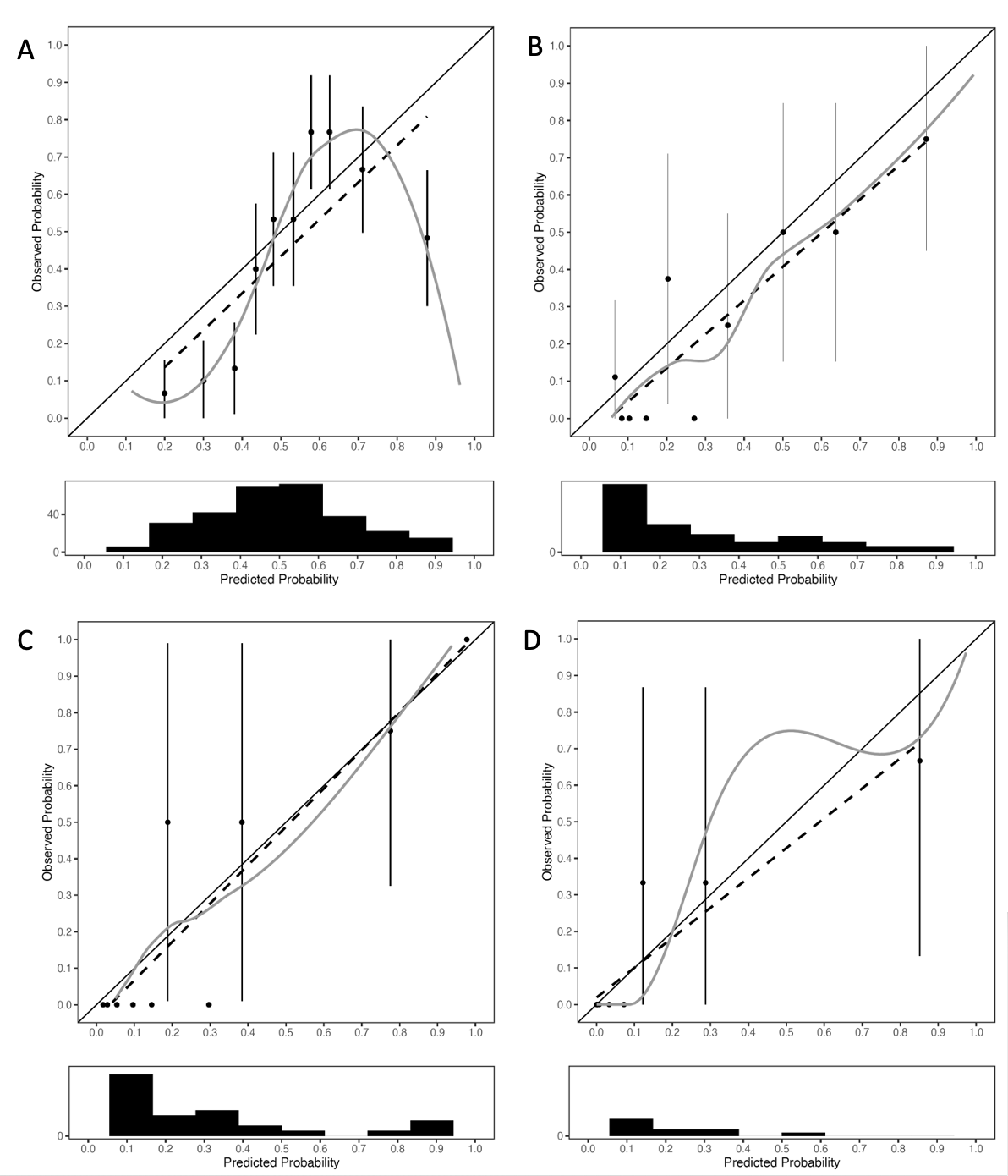


**Figure S2.** Receiver Operating Characteristic (ROC) curves for the mortality prognostic model sensitivity analysis using iterative biomarker data are shown with the base model including age and Ct (A), treatment day 1,2 (D_1,2_) model (B), treatment day 3,4 (D_3,4_) model (C), and treatment day 5,6 (D_5,6_) model (D). The ROC curve is plotted (central black line) with the 95% confidence interval band (grey shaded area).

**
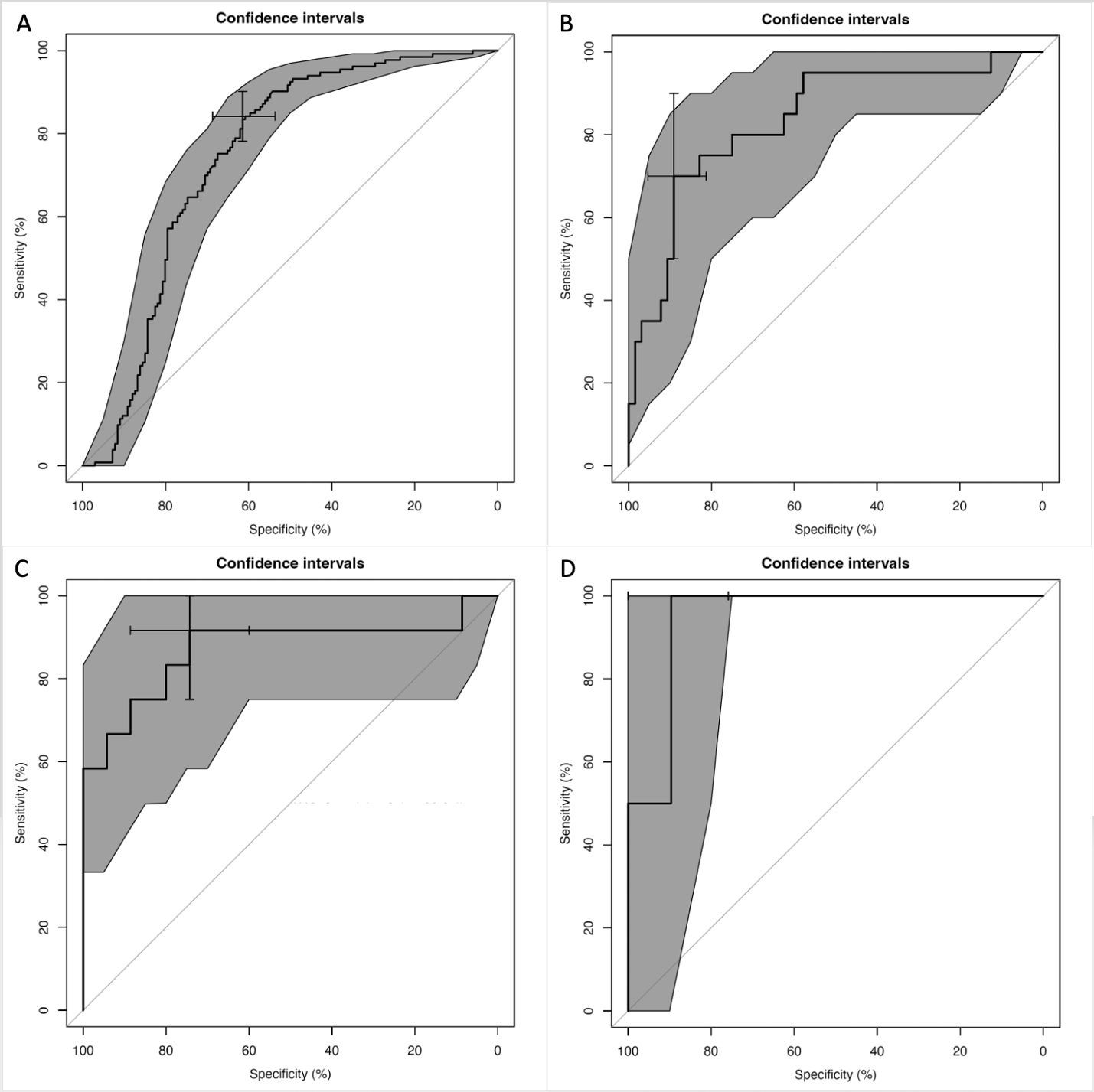
**

AUC 0.95 (95% CI 0.87-1.0)

AUC 0.87 (95% CI 0.72 – 1.0)

AUC 0.84 (95% CI 0.73 – 0.94)

AUC 0.74 (95% CI 0.69 – 0.80)

**Figure S3.** Calibration curves for the mortality prognostic model sensitivity analysis using iterative biomarker data for base model including age and Ct (A), treatment day 1,2 (D_1,2_) model (B), treatment day 3,4 (D_3,4_) model (C), and treatment day 5,6 (D_5,6_) model (D). The interpretation of the plots is the same as in Supplementary Figure 1.


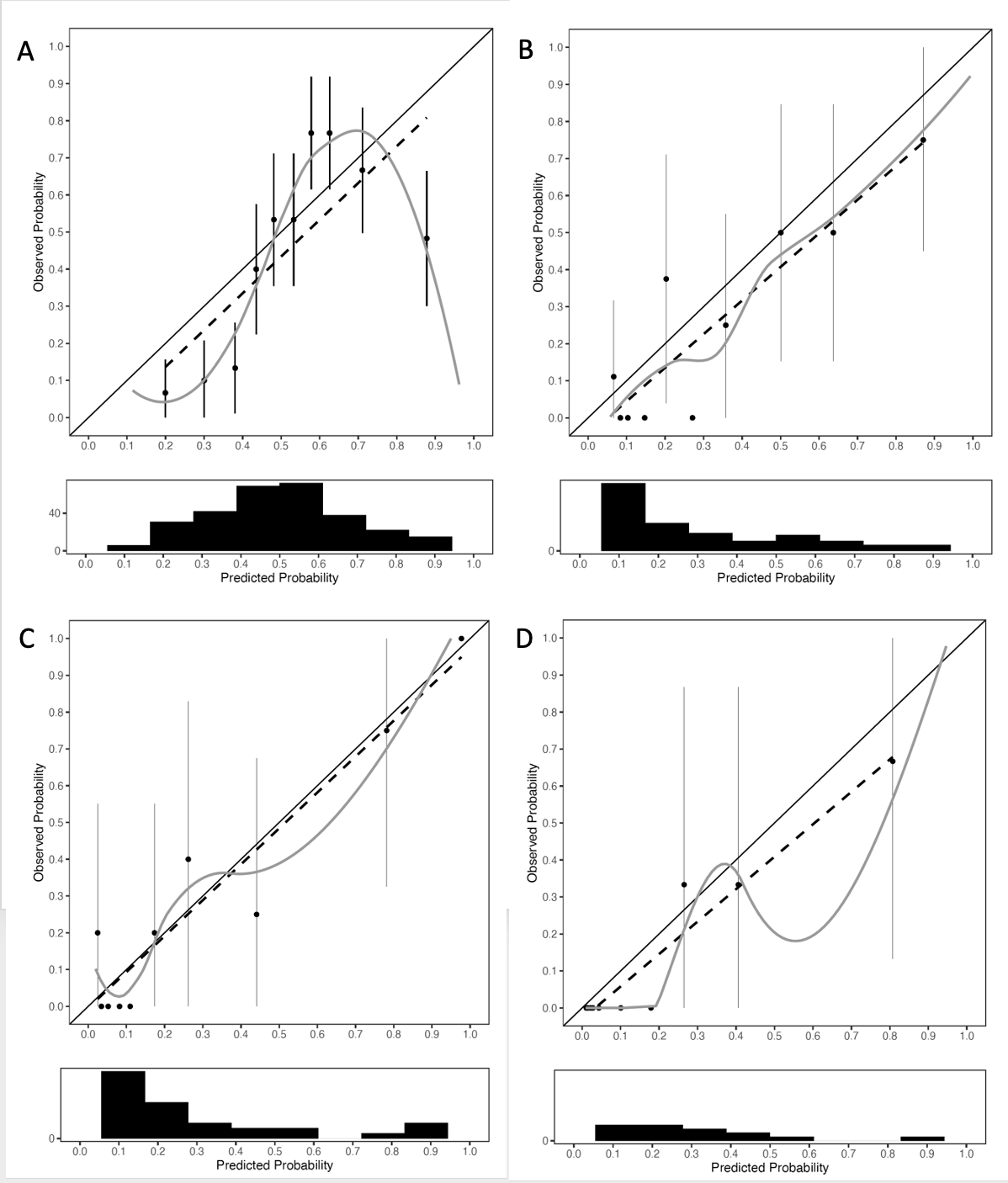


**Table S2**. Cohort characteristics of all patients and of patients with biomarker data available

|  | All patients | Biomarker data available | |  |
| --- | --- | --- | --- | --- |
| Variable | N = 310 (%) | Yes (N = 167) | No (N = 143) | p-value |
| Sex |  |  |  |  |
| Male | 134 (43.2) | 93 (55.7) | 60 (42.0) | 0.677 |
| Female | 176 (56.8) | 74 (44.3) | 83 (58.0) |  |
| Age [years], median (IQR) | 32 (24-46) | 33 (25 - 47) | 30 (23-45) | 0.382 |
| Vaccinated with rVSV-ZEBOV vaccine | 135 (44.0) | 79 (47.6) | 56 (39.7) | 0.228 |
| Time symptom onset to presentation [days], median (IQR) | 4 (2-6) | 4 (2-6) | 4 (2-7) | 0.01 |
| Symptoms reported at Admission^a^ |  |  |  |  |
| Fatigue | 260 (84.1) | 138 (82.6) | 122 (85.9) | 0.431 |
| Anorexia | 232 (74.8) | 120 (71.9) | 112 (79.3) | 0.191 |
| Subjective fever | 218 (70.3) | 121 (72.5) | 97 (67.8) | 0.047 |
| Headache | 208 (67.3) | 109 (65.3) | 99 (69.7) | 0.406 |
| Arthralgia | 195 (62.9) | 105 (62.9) | 90 (62.9) | 0.991 |
| Abdominal pain | 177 (57.1) | 93 (55.7) | 84 (58.7) | 0.588 |
| Myalgia | 175 (56.5) | 96 (57.5) | 79 (55.2) | 0.692 |
| Nausea | 153 (49.4) | 89 (53.3) | 64 (44.8) | 0.134 |
| Conjunctivitis | 150 (48.5) | 89 (53.3) | 61 (43.0) | 0.07 |
| Diarrhea | 149 (48.2) | 85 (50.9) | 64 (45.1) | 0.307 |
| Chest pain | 113 (36.6) | 63 (37.7) | 50 (35.2) | 0.648 |
| Cough | 84 (27.2) | 50 (29.9) | 34 (24.0) | 0.238 |
| Dysphagia | 67 (21.6) | 33 (19.8) | 34 (23.8) | 0.392 |
| Abnormal bleeding^b^ | 59 (19.1) | 29 (17.4) | 30 (21.1) | 0.402 |
| Sore throat | 57 (18.5) | 30 (18.0) | 27 (19.0) | 0.813 |
| Dyspnea | 48 (15.5) | 23 (13.8) | 25 (17.6) | 0.354 |
| Hiccup | 17 (5.5) | 13 (7.8) | 4 (2.8) | 0.056 |
| Coma | 14 (4.5) | 8 (4.8) | 6 (4.2) | 0.812 |
| Rash | 13 (4.2) | 8 (4.8) | 5 (3.5) | 0.58 |
| Jaundice | 11 (3.6) | 5 (3.0) | 6 (4.2) | 0.56 |
| Confusion | 11 (3.6) | 5 (3.0) | 6 (4.2) | 0.56 |
| Eye pain | 8 (2.6) | 5 (3.0) | 3 (2.1) | 0.627 |

^a^Percentages may sum to >100% due to multiple reported symptoms per person

^b^Abnormal bleeding including hematochezia, bleeding gums, non-menstrual vaginal bleeding, hematemesis, and epistaxis were aggregated into a single variable.

**Figure S4.** Receiver Operating Characteristic (ROC) curves for the mortality prognostic model sensitivity analysis using iterative biomarker data are shown with the base model including age and Ct (A), treatment day 1,2 (D_1,2_) model (B) including blood urea nitrogen (BUN), albumin, and alanine transaminase, treatment day 3,4 (D_3,4_) model (C) including BUN. The treatment day 5,6 (D_5,6_) model had too few subjects to perform the analysis. The ROC curve is plotted (central black line) with the 95% confidence interval band (grey shaded area).

**
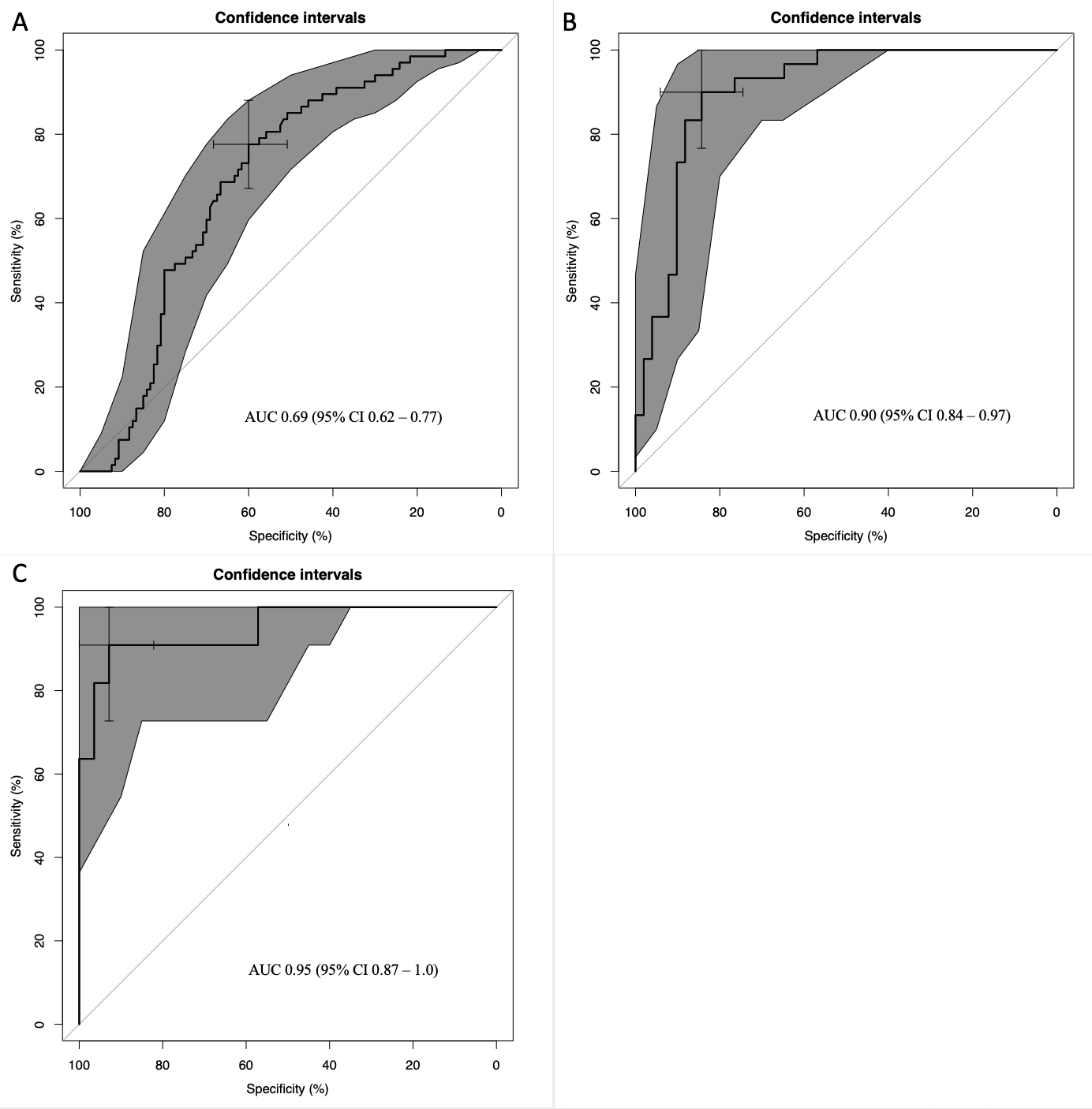
**
