# Supplementary material for: Derivation and Internal Validation of a Mortality Prognostication Machine Learning Model in Ebola Virus Disease Based on Iterative Point-of-Care Biomarkers
Source: Open Forum Infect Dis. 2024 Jan 5;11(2):ofad689. doi: 10.1093/ofid/ofad689 (PMC10878059; doi:10.1093/ofid/ofad689)
Supplement: ofad689_Supplementary_Data [file ofad689_supplementary_data.zip › Table S1.docx]

**Table S1.** Biomarker summary statistics during Ebola Treatment Center treatment day_1,2_, treatment day_3,4_, and treatment day_5,6_

|  |  | **Treatment days _1,2_** | | | **Treatment days _3,4_** | | | | **Treatment days _5.6_** | | | |
| --- | --- | --- | --- | --- | --- | --- | --- | --- | --- | --- | --- | --- |
| **Biomarker** |  | **Deceased**  **Median (IQR)** | **Survived Median (IQR)** | **p-value** |  | **Deceased Median (IQR)** | **Survived Median (IQR)** | **p-value** |  | **Deceased Median (IQR)** | **Survived Median (IQR)** | **p-value** |
| Blood Urea Nitrogen (mg/dL) |  | 51 (60) | 10 (18) | <0.0001 |  | 50.5 (57) | 8 (6) | <0.0001 |  | 42 (60) | 7 (9) | 0.0009 |
| Creatinine (mg/dL) |  | 3.7 (4.5) | 1 (1.1) | <0.0001 |  | 5.1 (5.1) | 0.9 (0.4) | <0.0001 |  | 6.2 (7.9) | 0.8 (0.5) | 0.0003 |
| Total bilirubin (mg/dL) |  | 1.1 (1.2) | 0.5 (0.3) | <0.0001 |  | 1.3 (1.3) | 0.5 (0.3) | <0.0001 |  | 1 (0.8) | 0.6 (0.4) | 0.075 |
| Albumin (g/dL) |  | 2.4 (0.7) | 2.8 (0.6) | 0.0009 |  | 1.8 (0.6) | 2.5 (0.6) | <0.0001 |  | 1.8 (0.6) | 2.4 (0.7) | 0.0006 |
| Aspartate transferase (U/L) |  | 1155 (1702) | 215.5 (537) | 0.011 |  | 954 (1658) | 283 (944.5) | 0.011 |  | 531 (511) | 219 (440) | 0.11 |
| Alanine transaminase (U/L) |  | 461.5 (699) | 70.5 (229) | <0.0001 |  | 569.5 (644) | 102.5 (239) | <0.0001 |  | 164.5 (288) | 137 (149) | 0.29 |
| Amylase (U/L) |  | 108.5 (97) | 86 (59) | 0.11 |  | 139 (230) | 89 (49) | 0.001 |  | 133 (3902) | 154.5 (3912) | 0.85 |
| Creatinine Kinase (U/L) |  | 1196 (1612) | 331 (535) | <0.0001 |  | 1871 (2588) | 426 (919.5) | <0.0001 |  | 1298 (1244) | 474.5 (562) | 0.0017 |
| C-reactive protein (mg/L) |  | 81.5 (88.9) | 31.7 (70.2) | 0.0009 |  | 111 (129.1) | 52.1 (89.3) | 0.0008 |  | 133.5 (141.8) | 32.6 (76.2) | 0.0014 |
|  |  | **Freq (%)** | **Freq (%)** | **p-value** |  | **Freq (%)** | **Freq (%)** | **p-value** |  | **Freq (%)** | **Freq (%)** | **p-value** |
| **Sodium (mmol/L)** |  |  |  |  |  |  |  |  |  |  |  |  |
| Hyponatremia |  | 6 (15.8) | 9 (15.8) | 0.47 |  | 2 (6.3) | 3 (6.3) | 0.71 |  | 1 (5.6) | 2 (4.0) | 0.071 |
| Eunatremia |  | 31 (81.6) | 48 (84.2) |  |  | 30 (93.8) | 44 (91.7) |  |  | 14 (77.8) | 47 (94.0) |  |
| Hypernatremia |  | 1 (2.6) | 0 (0.0) |  |  | 0 (0.0) | 1 (2.1) |  |  | 3 (16.7) | 1 (2.0) |  |
| **Potassium (mmol/L)** |  |  |  |  |  |  |  |  |  |  |  |  |
| Hypokalemia |  | 8 (25.8) | 19 (33.3) | 0.0051 |  | 7 (25.9) | 10 (21.7) | 0.0019 |  | 6 (37.5) | 13 (26.0) | 0.067 |
| Eukalemia |  | 16 (51.6) | 37 (64.9) |  |  | 10 (37.0) | 33 (71.7) |  |  | 7 (43.8) | 35 (70.0) |  |
| Hyperkalemia |  | 7 (22.6) | 1 (1.8) |  |  | 10 (37.0) | 3 (6.5) |  |  | 3 (18.8) | 2 (4.0) |  |
| **Calcium (mg/dL)** |  |  |  |  |  |  |  |  |  |  |  |  |
| Hypocalcemia |  | 22 (56.4) | 17 (29.3) | 0.024 |  | 23 (69.7) | 8 (16.7) | <0.0001 |  | 9 (50.0) | 13 (26.0) | 0.081 |
| Eucalcemia |  | 16 (41.0) | 40 (69.0) |  |  | 10 (30.3) | 40 (83.3) |  |  | 9 (50.0) | 37 (74.0) |  |
| Hypercalemia |  | 1 (2.6) | 1 (1.7) |  |  | 0 (0.0) | 0 (0.0) |  |  | 0 (0.0) | 0 (0.0) |  |
| **Glucose (mg/dL)** |  |  |  |  |  |  |  |  |  |  |  |  |
| Hypoglycemia |  | 14 (36.8) | 7 (12.1) | 0.011 |  | 14 (42.4) | 8 (16.7) | 0.0011 |  | 9 (50.0) | 9 (18.0) | 0.025 |
| Euglycemia |  | 16 (42.1) | 28 (48.3) |  |  | 9 (27.3) | 33 (68.8) |  |  | 6 (33.3) | 32 (64.0) |  |
| Hyperglycemia |  | 8 (21.1) | 23 (39.7) |  |  | 10 (30.3) | 7 (14.6) |  |  | 3 (16.7) | 9 (18.0) |  |
